# Esophageal Intubation of an Infant

**DOI:** 10.5811/westjem.2015.4.25821

**Published:** 2015-06-17

**Authors:** Jana L. Anderson, Kharmene Sunga, Annie Sadosty

**Affiliations:** Mayo Clinic, Department of Emergency Medicine, Rochester, Minnesota

A 68-day-old former 30-week infant presented with listlessness, apnea and bradycardia. The patient was intubated for airway protection. After intubation, breath sounds were auscultated bilaterally and a Pedi-Cap carbon dioxide detector had color change from purple to yellow. A nasogastric tube (NGT) was placed and a post-procedural chest radiograph was obtained (Figure).

There are several features of esophageal intubation: low lung volumes, esophageal and gastric distention despite NGT placement and juxtaposition of the endotracheal tube (ETT) relative to the NGT.^1^–^2^ Other findings of esophageal intubation not seen here are identification of the ETT distal to the carina or outside of the tracheal-bronchial air column.^3^ Due to high success rates of endotracheal intubation in the emergency department,^4^–^5^ these findings are rare and may be overlooked. In this case, misleading clinical evidence was obtained through auscultation of bilateral breath sounds, visualization of endotracheal tube condensation, positive change on the carbon dioxide colorimeter and post-procedural hemodynamic and oxygenation stability. Previous literature, however, has demonstrated false-positive colorimetric change from swallowed air with pre-intubation positive pressure ventilation,^6^–^7^ hence the importance of radiographic identification of ETT location. In this patient, esophageal intubation was recognized after continuous capnography revealed absence of waveform.

## Figures and Tables

**Figure:**
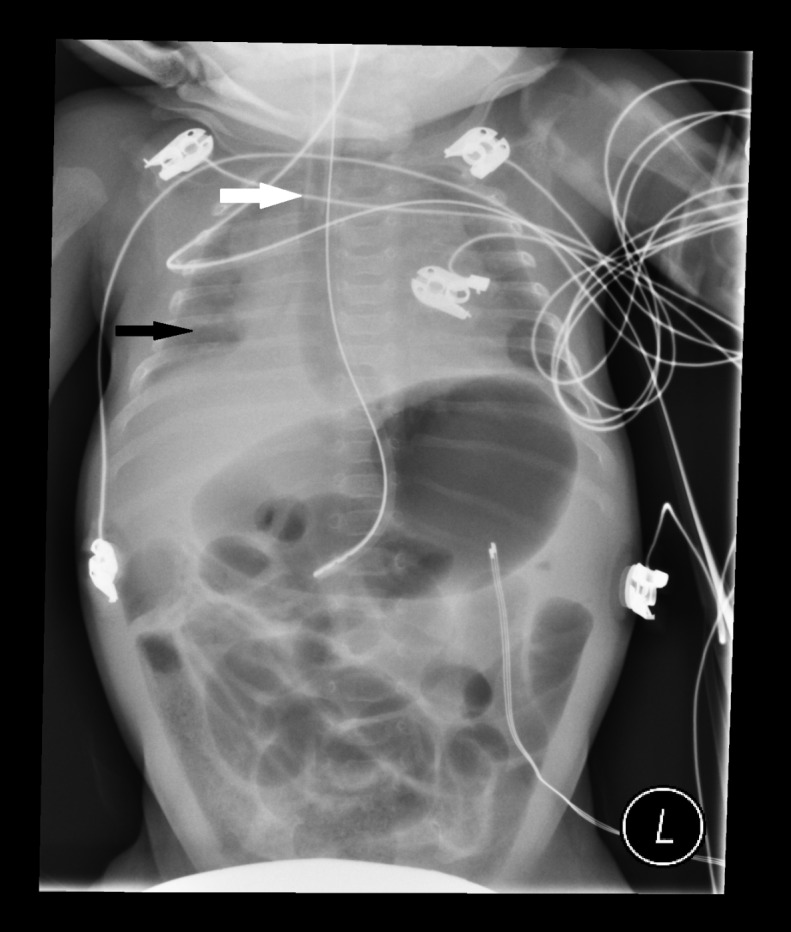
Infant with endotracheal tube in the esophagus; nasogastric tube present in the stomach. White arrow indicates endotracheal tube tip. Black arrow indicates low lung volumes.
